# Musical Hallucinations in an Elderly Female With Hearing Loss

**DOI:** 10.7759/cureus.72992

**Published:** 2024-11-04

**Authors:** Jacqueline Cavendish, Lynne J Goebel

**Affiliations:** 1 Internal Medicine, Joan C. Edwards School of Medicine at Marshall University, Huntington, USA; 2 Internal Medicine and Geriatrics, Joan C. Edwards School of Medicine at Marshall University, Huntington, USA

**Keywords:** auditory charles bonnet syndrome, hearing loss, musical ear syndrome, musical hallucinations, sensorineural hearing loss

## Abstract

Musical hallucinations (MH) are rare auditory phenomena in which patients perceive music without a known source. Risk factors for MH include hearing loss, older age, female gender, epilepsy, and certain medications. This case report details the presentation, diagnosis, and treatment of an 82-year-old woman who developed MH following the onset of hearing loss. Despite treatment attempts with gabapentin, quetiapine, and donepezil, the symptoms persisted, demonstrating the difficulty in treating this condition.

## Introduction

Musical hallucinations (MH), also known as musical ear syndrome or auditory Charles Bonnet syndrome, are rare auditory phenomena in which a patient perceives music or musical sounds without any actual music present. A recent review of MH manuscripts published between 2005 and 2022 revealed not otherwise classifiable (25%) followed by psychiatric disorders (23%) and hearing impairment (22%) as the most frequent etiologies [1]. Although hearing loss is a common reason for MH, when you examine cohorts of people with hearing loss, the phenomenon is quite rare. One study found that in 193 patients with mild to severe hearing loss, only 3.6% of patients experienced MH [2]. MH can also arise from various neurological, psychiatric, or pharmacological conditions and are linked to older age, female gender, cochlear implants, and epilepsy [1,3-5].

Since the nineteenth century, doctors reported patients hearing familiar songs, musical sounds, or melodies [6]. The specific presentation of MH varied widely among patients. Some patients experienced continuous hallucinations while others had intermittent episodes. The impact on their daily lives ranged from minimal to significant, with some patients describing the symptoms as significantly distressing. The nature of the music also varied from patient to patient, with many patients hearing familiar music. The prevalence of MH is possibly underreported due to patients' reluctance to disclose their experiences or because the hallucinations are not particularly distressing. For example, in a series of people with cochlear implants and MH, only 11% found them intolerable [4].

In this case report, we describe an 82-year-old female with bilateral chronic serous otitis media and bilateral sensorineural hearing loss who experiences persistent MH. This report adds to the growing body of evidence linking auditory hallucinations to hearing impairment and highlights the need for awareness and further research into the etiology and treatment of MH.

## Case presentation

The patient is an 86-year-old female with a medical history notable for bilateral chronic serous otitis media, bilateral sensorineural hearing loss, post-traumatic stress disorder (PTSD), hypertension, stroke, trauma to the brain, and seizures. At 82 years of age, she developed a progressive worsening of her hearing loss and began experiencing MH. The patient initially attributed the music to her neighbors or other external sources. However, over time, she realized others could not hear the music. The patient described her auditory hallucinations as continuous, frequently familiar music playing in both of her ears at a normal volume. Occasionally, she heard unpleasant and unfamiliar music, like a broken record. She also noted that she could influence the music and change the tune playing by singing along. She frequently heard familiar pop music, such as songs by Frank Sinatra, but during the winter months, she heard Christmas tunes. She stated that she could still watch TV and did not find the hallucinations particularly distressing. Her regular medications included biotin, vitamin C, fish oil, multivitamin, vitamin D3, lisinopril, metoprolol, warfarin, and temazepam. She was fully independent in activities of daily living and lived alone.

**Figure 1:**
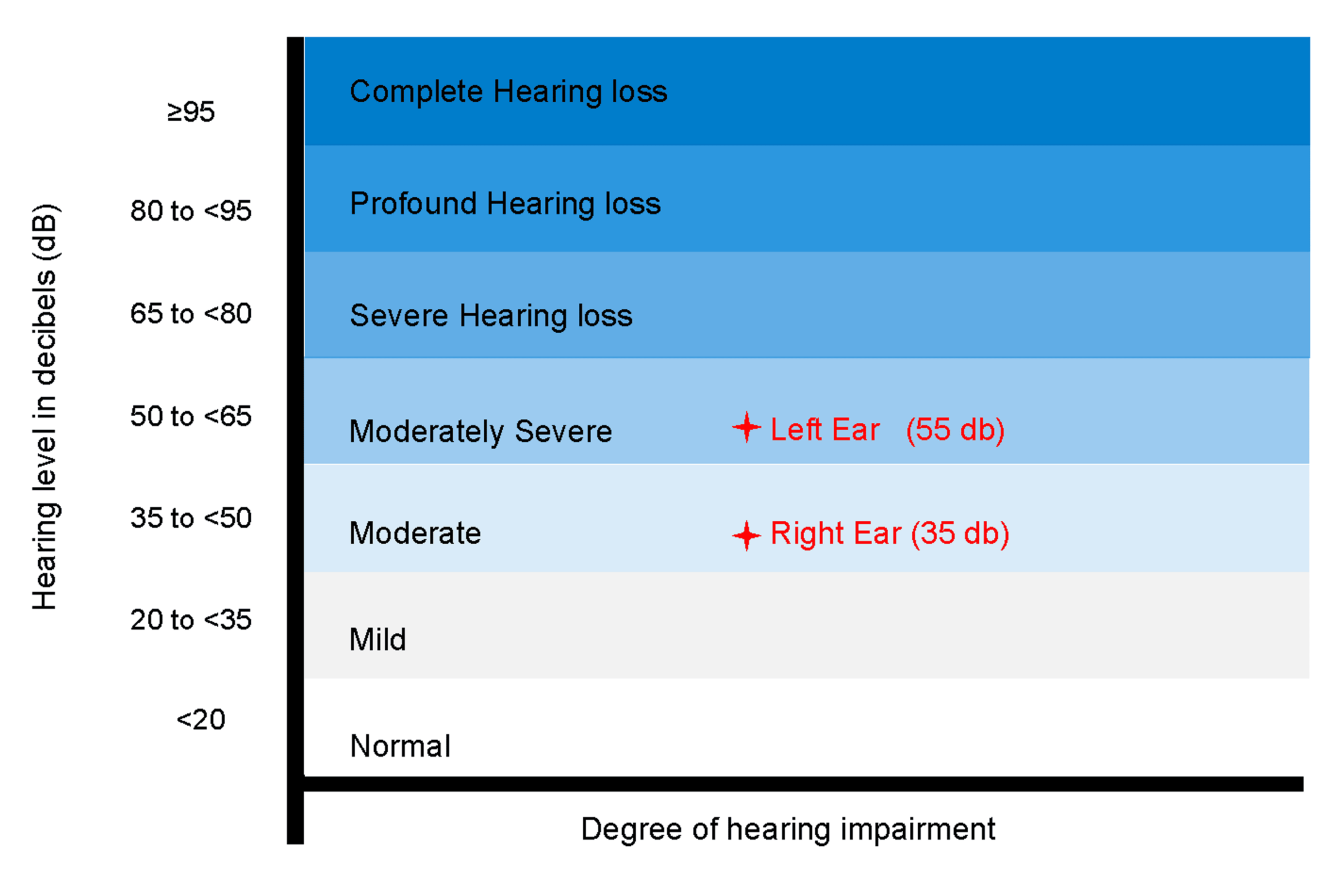
Audiogram depicting bilateral hearing loss severity

Initially, she consulted an otolaryngologist, who attributed the MH to hearing loss secondary to sensorineural impairment and serous otitis media. He used the speech reception threshold (SRT), which measures the quietest level at which a person can recognize and repeat back speech stimuli, to evaluate her hearing loss. The SRT values for this patient were 35 dB in the right ear (AD) and 55 dB in the left ear (AL), indicating moderate and moderately severe hearing loss, respectively (Figure 1) [7].

Unfortunately, hearing aids and pressure equalization tubes for the serous otitis media did not resolve the MH. Medication tried included gabapentin, quetiapine, and donepezil, with no noticeable improvement in her symptoms. After several years since the onset of her MH, the patient stated the music no longer bothered her, and she declined any further treatment.

## Discussion

Our patient has several possible etiologies for her MH, including hearing loss, stroke, seizures, and benzodiazepine use. Hearing loss accounts for a large portion of patients with MH, and we suggest that our patient's bilateral hearing loss likely played a significant role. A recent review found that MH most frequently occurs in elderly individuals, especially women, and commonly those with hearing impairment [1]. Other conditions linked to MH include medications, epilepsy, psychiatric conditions, stroke, and sometimes a combination of these things [1,3,5,8]. Specifically, a recent literature review identified several common medications associated with MH, including antidepressants, antiparkinsonian agents, opioids, N-methyl-D-aspartate (NMDA) antagonists, and benzodiazepines [9]. The authors proposed that these medications may induce MH by disrupting neurotransmitter balance, particularly involving dopamine and GABA pathways. Our patient took temazepam, a benzodiazepine known to be a potential trigger for MH. However, her symptoms of MH persisted even when she was not taking this medication, suggesting that temazepam alone does not fully account for her symptoms and indicates another underlying cause. Although our patient has a history of seizures, the onset of MH following her hearing loss, rather than after a seizure episode, suggests it is more likely related to her auditory impairment. The patient had not had a seizure in many years and unfortunately, we do not have her EEG to demonstrate the area of the brain involved. Her stroke, which was remote with no lasting deficits was also not associated temporally with the onset of MH and, therefore, thought to be unrelated.

The underlying pathophysiology of MH is unclear. In their 2020 study, Marschall et al. employed functional magnetic resonance imaging (fMRI) to investigate complex hallucinations, including speech and music, in 18 patients with hearing loss [10]. The researchers noted increased activity in the anterior and posterior cingulate cortex and bilateral temporal cortex. These findings implied a possible reduction in error monitoring in individuals with hearing impairments, supporting the deafferentation theory of MH. This theory suggests that reduced sensory input to the auditory centers of the brain lowers the threshold for detecting neural activity, leading to the misinterpretation of spontaneous neural activity as actual sounds and, consequently, producing hallucinations. Notably, Charles Bonnet syndrome is a condition in which patients experience visual hallucinations following vision loss, indicating that similar sensory deprivation mechanisms can cause hallucinations. This resemblance has led some to refer to MH as an auditory Charles Bonnet syndrome [11]. Further supporting this, Aldhafeeri et al. employed fMRI to examine MH in a patient with hearing loss, observing increased neural activity and reduced cortical thickness in the prefrontal, temporal, and limbic regions [12]. These anatomic changes suggest that hearing loss may contribute to altered neural activity, aligning with the deafferentation theory observed in Charles Bonnet syndrome. Overall, further research is needed to explore the complex interplay between sensory deprivation, brain activity, and the development of MH.

The treatment of MH primarily focuses on addressing the underlying cause such as hearing loss or psychosis. However, there is no specific preferred treatment designed solely to eliminate the hallucinations. Common management strategies include discontinuing any potential causative medications and starting antipsychotics [3]. Mansoor et al. described successful treatment with quetiapine in one case and olanzapine in two cases [13]. We prescribed quetiapine to our patient who reported help with sleep but not with MH. A review by Colon-Rivera and Oldham presented a compilation of case reports showing successful treatment with antipsychotics, donepezil, lamotrigine, gabapentin, and electroconvulsive therapy [14]. Unfortunately, we tried donepezil and gabapentin in our patient without success. Antipsychotics are thought to reduce symptoms of musical hallucinations by modulating neurotransmitter activity. Specifically, quetiapine acts by blocking dopamine and serotonin receptors while donepezil works by increasing acetylcholine levels. However, the exact mechanism by which these medications alleviate MH remains unclear, representing an important area for future research. In 2021, Nordberg et al. successfully treated one patient with MH using repetitive transcranial magnetic stimulation to the left temporal parietal junction [15]. Improving auditory input through hearing aids or cochlear implants can also help alleviate MH symptoms, though cochlear implants are also associated with the onset of MH [4]. However, further studies are needed to confirm the effectiveness of these approaches in other patients. Overall, the best treatment and management of MH remains unknown, and as the condition is uncommon and likely multifactorial, it is not conducive to study in randomized clinical trials.

## Conclusions

This case report highlights the complex nature of MH and contributes to the growing literature on the relationship between MH and sensorineural hearing loss. Additionally, it emphasizes the need for further research into the pathophysiology and treatment of MH, as current understanding is limited and therapies are variably successful. The complex interplay between auditory processing and neural networks in the manifestation of MH could be important in developing an effective treatment for this condition. Our case also demonstrates the variable presentation of MH and its impact on patients' lives. For example, our patient was only bothered by MH when the music was not enjoyable, and ultimately, she did not require treatment, as she adapted to the MH. We hope this case describes the challenge of MH as a consequence of hearing impairment and the need for better management strategies in affected patients who are bothered by the symptoms.
